# Equine Mesenchymal Stem Cells Influence the Proliferative Response of Lymphocytes: Effect of Inflammation, Differentiation and MHC-Compatibility

**DOI:** 10.3390/ani12080984

**Published:** 2022-04-11

**Authors:** Alina Cequier, Antonio Romero, Francisco J. Vázquez, Arantza Vitoria, Elvira Bernad, Sara Fuente, Pilar Zaragoza, Clementina Rodellar, Laura Barrachina

**Affiliations:** 1 Laboratorio de Genética Bioquímica LAGENBIO, Instituto de Investigación Sanitaria de Aragón (IIS), Universidad de Zaragoza, C/Miguel Servet 177, 50013 Zaragoza, Spain; alinacs@unizar.es (A.C.); aromerol@unizar.es (A.R.); pvazquez@unizar.es (F.J.V.); avm@unizar.es (A.V.); ebernadroche@gmail.com (E.B.); sfuente@unizar.es (S.F.); pilarzar@unizar.es (P.Z.); lbarrach@unizar.es (L.B.); 2 Instituto Agroalimentario de Aragón-IA2, Universidad de Zaragoza, C/Miguel Servet 177, 50013 Zaragoza, Spain; 3 Servicio de Cirugía y Medicina Equina, Hospital Veterinario, Universidad de Zaragoza, C/Miguel Servet 177, 50013 Zaragoza, Spain

**Keywords:** horse, mesenchymal stem cells, immunomodulation, immunogenicity, immunosuppression assay, modified mixed leukocyte reaction, allogenic therapy, flow cytometry, co-culture, haplotype

## Abstract

**Simple Summary:**

Mesenchymal stem cells are investigated for therapy because of their ability to regulate the immune response to an injury. Cell therapy is increasingly important in veterinary patients such as horses, which are also valuable as a model. Therefore, what is learned in these animals can benefit both them and people. However, the patient’s immune system could recognize and destroy mesenchymal stem cells, impairing effectiveness and potentially leading to adverse effects. In this study, we analysed how equine mesenchymal stem cells interact with immune cells in different scenarios. We tested the effect of inflammation and differentiation of these cells, and how they acted depending on donor–patient compatibility. As we expected, inflammation activated the regulatory ability of equine mesenchymal stem cells, but also increased the risk of immune recognition. We anticipated that, after differentiation, these cells would lose their regulatory ability and would be more easily targeted by the immune system. However, they maintained similar features after differentiating into cartilage cells. The balance between the ability of mesenchymal stem cells to stimulate and to regulate an immune response is of the utmost importance to develop safe and effective cell therapies for animals and people.

**Abstract:**

Immunomodulation and immunogenicity are pivotal aspects for the therapeutic use of mesenchymal stem cells (MSCs). Since the horse is highly valuable as both a patient and translational model, further knowledge on equine MSC immune properties is required. This study analysed how inflammation, chondrogenic differentiation and compatibility for the major histocompatibility complex (MHC) influence the MSC immunomodulatory–immunogenicity balance. Equine MSCs in basal conditions, pro-inflammatory primed (MSC-primed) or chondrogenically differentiated (MSC-chondro) were co-cultured with either autologous or allogeneic MHC-matched/mismatched lymphocytes in immune-suppressive assays (immunomodulation) and in modified one-way mixed leukocyte reactions (immunogenicity). After co-culture, frequency and proliferation of T cell subsets and B cells were assessed by flow cytometry and interferon-ɣ (IFNɣ) secretion by ELISA. MSC-primed showed higher regulatory potential by decreasing proliferation of cytotoxic and helper T cells and B cells. However, MHC-mismatched MSC-primed can also activate lymphocytes (proliferative response and IFNɣ secretion), likely due to increased MHC-expression. MSC-chondro maintained their regulatory ability and did not increase their immunogenicity, but showed less capacity than MSC-primed to induce regulatory T cells and further stimulated B cells. Subsequent in vivo studies are needed to elucidate the complex interactions between MSCs and the recipient immune system, which is critical to develop safe and effective therapies.

## 1. Introduction

Mesenchymal stem cells (MSCs) are adult multipotent stem cells that have raised interest in the field of regenerative medicine due to their unique biological properties [1]. Mesenchymal stem cells have potential for treating several pathologies, such as those affecting the musculoskeletal system. However, MSC paracrine activity, including immunomodulatory effects, have broadened their scope for inflammatory and immune-mediated pathologies. As the etiopathogenesis of some diseases is similar in both people and animals, the results obtained can be valuable for both human and veterinary patients [2]. The horse presents a remarkable value for translational medicine [3], but further research on the mechanisms of equine MSC, especially their immune properties, is needed. The immune regulatory mechanisms of MSCs mainly depend on their paracrine activity [4], which is not only linked to their therapeutic effects, but also to their ability to escape from immune recognition when administered allogenically. Actually, a highly relevant paradigm change is that MSCs are not truly immune-privileged but immune-evasive, and thus, their recognition and elimination by the immune system in the allogeneic setting should be considered [5]. Allogeneic application presents several advantages over autologous therapy, including the possibility of providing quicker and wider availability of well-characterized MSCs, and its application when autologous cells are not appropriate [6].

Therefore, MSC immunogenicity should be further investigated to develop safe and effective treatments. Cellular and humoral immune responses may be generated against allogeneic MSCs, thus limiting their effects and potentially leading to adverse reactions [7,8]. Furthermore, immune memory mechanisms could be developed and compromise the repeated administration of allogeneic MSCs in the horse [9]. In recent years, the importance of the major histocompatibility complex (MHC) matching between donor and recipient has been studied by several authors in the horse [7,10], as well as in other species [11,12]. As the therapeutic effectiveness could be compromised by the generation of antibodies specifically directed against the equine leukocyte antigen (ELA) of the donor, ELA haplotypes and compatibility should be taken into account.

In addition, it has also been reported that the MHC level expression in MSCs in basal conditions is quite dependent on the equine donor [7]. However, there are other factors that may modify the initial immune properties of MSCs, such as their exposure to an inflammatory environment and the process of differentiation. The knowledge on these factors is critical to use them for designing more effective therapeutic strategies.

Regarding inflammatory exposure, priming equine MSCs with pro-inflammatory cytokines like interferon gamma (IFNγ) and tumour necrosis factor alpha (TNFα) increases their immunomodulatory properties and may result in enhanced regulatory effects in vivo [13]. However, priming MSCs may also raise their immunogenicity because of induced expression of MHC, thus potentially limiting the allogeneic administration [14]. The balance between immunomodulation and immunogenicity induction seems to depend on the type of inflammatory exposure. For example, high doses of interferon (IFNγ) appears to increase the expression of MHC-II [7], whereas a short priming of equine MSCs with low doses of IFNγ and TNFα could maintain the balance between immunomodulatory and immunogenic factors [15]. It has also been suggested that MSCs may lose their regulatory abilities and increase their immunogenicity after differentiation [16]. The increased expression of MHC-I and/or MHC-II could result in a higher risk of allo-recognition if equine MSCs differentiate after administration or are administered pre-differentiated [17], especially if their regulatory abilities are also impaired by differentiation.

In spite of all the aforementioned, allogeneic MSC administration has been shown to be safe and effective in several conditions [10,18,19]. Therefore, it has been suggested that the balance between their immunomodulatory and immunogenic potentials would allow allogeneic MSCs to evade the immune system and elicit their actions [14]. To strengthen the development of allogeneic cell therapies, it is critical to gain knowledge into how factors such as MHC matching/mismatching, inflammation and differentiation may affect such balance in equine MSCs. To the best of authors’ knowledge, there are not studies simultaneously assessing these factors on equine MSCs.

Therefore, the aim of this study was to analyse the changes elicited in vitro by equine MSCs on relevant lymphocyte subpopulations under different conditions: inflammation, differentiation, and compatibility for the MHC. For this purpose, lymphocyte proliferation assays consisting of immunosuppression assays (i.e., immunomodulatory capacity) and modified one-way mixed leukocyte reactions (MLRs) (i.e., immunogenic potential) were carried out using equine MSCs in basal conditions (MSC-naïve), pro-inflammatory primed (MSC-primed) or chondrogenically differentiated (MSC-chondro), co-cultured with either autologous or allogeneic MHC-matched/mismatched lymphocytes. The initial hypothesis was that inflammatory priming would markedly increase MSC immune regulatory potential with a slight effect on MSC immunogenicity, whereas differentiation would diminish their immunomodulatory ability and increase their immunogenicity. Regarding the MHC compatibility, it was hypothesized that MHC-matched cells would generate similar results than autologous ones, whereas the MHC-mismatched co-cultures would result in similar immunomodulation but increased immunogenicity.

## 2. Materials and Methods

### 2.1. Study Design

**Figure 1:**
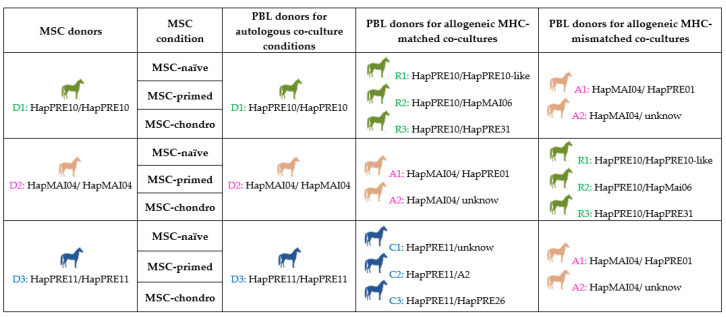
Study design showing mesenchymal stem cells (MSCs) and peripheral blood lymphocytes (PBLs) donors to establish autologous and allogeneic major histocompatibility complex (MHC)-matched and MHC-mismatched co-cultures, to study immunomodulatory capacity and immunogenic potential. MSCs were assayed unstimulated (MSC-naïve), primed with cytokines (MSC-primed) or chondrogenically differentiated (MSC-chondro).

Three MHC homozygous horses were used as donors of bone marrow derived MSCs to study their immunomodulatory capacity and immunogenic potential in three conditions: basal (MSC-naïve), proinflammatory-primed (MSC-primed) and chondrogenically-differentiated (MSC-chondro) conditions. For this purpose, MSCs under the different conditions were co-cultured with peripheral blood lymphocytes (PBLs) autologous (*n* = 3) or allogeneic from MHC-matched (*n* = 8) and mismatched (*n* = 7) animals. A total of 11 animals, selected by their MHC haplotype, were involved for MSCs and PBLs collection and co-cultures were established using the combinations shown in Figure 1. Two types of co-cultures were used: immunosuppressive assays to study MSC immunomodulatory potential, and one-way modified MLR to assess MSC immunogenicity. PBLs were stained with carboxyfluorescein succinimidyl ester (CFSE) to evaluate their proliferation after each type of co-culture. In addition, at the end of the co-culture assays, PBLs were stained with a panel of antibodies to study changes in the frequency and proliferation of each lymphocyte subset by flow cytometry. The secretion of IFNγ in co-culture supernatants was assessed by ELISA as a reflection of T cell activation.

### 2.2. Animal Selection by MHC-Haplotyping

Eleven mixed-breed horses (1 stallion, 3 geldings, 7 mares; aged 2–8 years, weight 412–493 kg) in good health status and with no previous pregnancy history were chosen based on their MHC haplotypes. Haplotypes were determined by microsatellite typing using a validated panel of 10 highly polymorphic intra-MHC regions, as previously described [9,20]. To find and select animals, a screening of 60 Purebred Spanish and mixed-breed horses from a local farm was performed. Blood was collected with informed owner’s consent and methodology for DNA extraction, multiplex PCRs and fragment analysis was performed as previously reported by our group [9].

Definitive haplotypes were established for homozygous animals and the remaining animals were assigned with provisional haplotypes based on previously known ones, either reported in the bibliography [10,21], or described in a preliminary study of our group in Purebred Spanish horses [22]. Three groups of animals were selected, including one a homozygous horse for each of the haplotypes HapPRE10, HapPRE11 or HapMAI04, and two to three heterozygous animals sharing one haplotype. In each group, the homozygous horse served as a MSC donor as it was MHC-matched with the heterozygous animals in the group, but MHC-mismatched with animals in other groups, thus allowing autologous, matched and mismatched combinations (Figure 1). The premise of using homozygous individuals for matching with different heterozygous individuals is the same proposed to create haplo-banks of human induced pluripotent stem cells (iPSCs) [23,24], and also used in equine MSC studies [25]. Table 1 shows the microsatellite alleles of each haplotype identified in the different horses chosen.

All the procedures involving animals were carried out under the Project License PI 15/16 approved by the in-house Advisory Ethics Committee for Animal Research from the University of Zaragoza. The care and use of animals were performed accordingly with the Spanish Policy for Animal Protection RD53/2013, which meets the European Union Directive 2010/63. All animals were kept on paddocks of the facilities of the Animal Research Service of the University of Zaragoza, with free access to water and fed with ad libitum grass hay.

### 2.3. Isolation and Characterization of MSCs

Equine bone marrow MSCs were obtained as previously described [15,17]. Briefly, bone marrow was harvested from the sternum of D1, D2 and D3 animals under sedation (0.04 mg/kg IV romifidine; Sedivet, Boehringer-Ingelheim, Barcelona, Spain and 0.02 mg/kg IV butorphanol; Torbugesic, Pfizer, Madrid, Spain) and local analgesia with lidocaine (Anesvet, Laboratorios Ovejero, León, Spain). Mononuclear cells were separated by density gradient centrifugation and seeded in culture medium consisting of low-glucose Dulbecco’s modified Eagle’s medium supplemented with 2 mM L-glutamine, 0.1 mg/mL streptomycin, 100 U/mL penicillin and 10% foetal bovine serum (FBS) (all from Sigma-Aldrich, Madrid, Spain). Cells were expanded until passage three, and then cryopreserved in 10% DMSO (Sigma-Aldrich) and 90% FBS medium until subsequent experiments started.

Cells were characterized at passage three by their phenotype and tri-lineage differentiation, as previously described [15,17,27] according to minimal criteria for defining human MSCs established by the International Society for Cellular Therapy [28]. Details on the methodology used and characterization data obtained are provided in Supplementary Materials S1.

Surface expression of MHC-I and MHC-II was studied by flow cytometry in both MSC-naïve and MSC-primed, since inflammatory exposure can induce changes in MHC expression. Methodology followed and antibody suitability was previously described [13,29], and is briefly explained in Supplementary Materials S1.

#### Preparation of MSC Experimental Conditions: MSC-Naïve, MSC-Primed and MSC-Chondro

Cryopreserved MSCs (*n* = 3) were thawed and seeded at 5000 cells/cm^2^ in the basal medium described above, at 37 °C and 5% CO_2_ for 72 h to recover from freezing. Subsequently, MSCs were detached with 0.25% trypsin-EDTA (Sigma–Aldrich) and seeded into a 24-well plate at 100,000 cells per well for the immunomodulatory assays, and at 20,000 MSCs per well for the immunogenicity assays. For MSC-naïve and MSC-primed, plating was done 24 h prior to co-cultures to allow MSCs to attach to the well. For MSC-chondro, plating was performed with the same amount of cells, but 14 days before the co-cultures to induce differentiation.

For the MSCs-primed condition, corresponding MSCs were exposed for 12 h to 5 ng/mL of equine recombinant TNFα (R&D Systems, Barcelona, Spain) plus 5 ng/mL of equine recombinant IFNγ (R&D Systems), added to the basal culture medium described above [15], before adding PBLs.

Chondrogenic differentiation for the MSC-chondro condition was induced with the StemPro™ Chondrogenesis Differentiation Kit (Thermo Fisher, Madrid, Spain) using the micro-mass system during 14 days [30], before adding PBLs. The protocol was adapted to suit the required amount of MSCs for the ratios selected for immunomodulatory and immunogenicity assay, which were 100,000 and 20,000 MSCs per well, respectively. For each donor, two replicates of each MSC type were prepared to run each co-culture in duplicate. Wells were washed with phosphate buffered saline (PBS, Gibco, Thermo Fisher), before adding PBLs.

### 2.4. Co-Cultures for Lymphocyte Proliferation Assays: Immunosuppression and Modified One-Way MLR

#### 2.4.1. Optimization of the Assays: CFSE Staining and Mitogen Stimulation

Prior to any experimental assay, the optimal concentration of CFSE (Sigma-Aldrich) and PBLs for staining were determined. Two different CFSE concentrations (2.5 μM and 5 μM) and two PBL concentrations (10 × 10^6^ PBL/mL and 20 × 10^6^ PBL/mL) were examined based on previously reported conditions [31]. After testing all the combinations, using 2.5 μM CFSE to stain cells at 20 × 10^6^ PBL/mL were set as the most appropriate conditions. Subsequently, a gradient of concentrations of the mitogen phytohemagglutin isoform P (PHA, Sigma-Aldrich) (2.5, 5, 10, and 20 μg/mL) were tested according to previous reports [32] and assessed after 2, 3 and 4 days [33,34]. The combination of PHA concentration and time of stimulation that provided maximal proliferation was 10 μg/mL of PHA during 3 days, and as such was set for all the immunomodulatory assays.

#### 2.4.2. Blood Collection and Isolation of PBLs

Peripheral blood lymphocytes were isolated using the carbonyl iron granulocyte depletion method, followed by density gradient centrifugation with Lymphoprep^TM^ (Fisher Scientific, Madrid, Spain) as previously described [8,9]. Briefly, blood was collected aseptically via jugular venipuncture into sterile 60-mL syringes with 17 I.U./mL of lithium heparin (Sigma-Aldrich), and plasma was allowed to separate for 20′ at room temperature (RT).

Plasma was separately collected into conical tubes using extension sets and incubated with carbonyl iron (Sigma-Aldrich) in agitation for 30′ at 37 °C. Then, carbonyl iron was placed in the bottom of the tubes by using a magnet, and supernatant was collected and centrifuged at 310× *g* 5′. The cellular pellet was resuspended in PBS and overlayed on Lymphoprep^TM^. After 690× *g* 15′ centrifugation (without brake), a lymphocyte layer was recovered and washed with PBS. Cells were counted in a hemocytometer chamber using Trypan Blue 0.4% as dye exclusion, and concentration was adjusted to 10 × 10^6^ live cells/mL in PBS. This isolation technique has been reported to provide an enriched lymphocyte population (95–99%) [9,35].

#### 2.4.3. Carboxyfluorescein Succinimidyl Ester Labelling and Analysis of Proliferation

Cells were labelled with 2.5 μM CFSE to measure lymphocyte proliferation by assessing CFSE dilution using flow cytometry [7,36]. After isolation, PBLs were placed in 15 mL conical tubes, centrifuged and resuspended at 20 × 10^6^ PBLs/mL in RPMI 1640 medium (Thermo Fisher), supplemented with 5% FBS to minimize cell toxicity, and 2.5 μM CFSE. Cells were evenly mixed to ensure rapid and homogeneous labelling and were incubated 5′ RT in dark [31]. The reaction was blocked by adding 2 mL of ice-cold FBS 1′ RT, and then the cells were washed once with PBS and twice with PBL medium (consisting of RPMI 1640 medium supplemented with 10% FBS, 0.1 mM 2-mercaptoethanol (Sigma), 100 U/mL penicillin, and 100 μg/mL streptomycin). Cells from the same horse but stained in different tubes were not mixed but used for separate immunomodulation or immunogenicity assays, to prevent heterogeneous intensity of the CFSE staining. After the staining procedure, PBLs were counted again and adjusted to 10 × 10^6^ live cells/mL in PBL medium.

#### 2.4.4. Immunosuppression Assay

The immunomodulatory function of MSCs was determined by their ability to modulate the proliferation of mitogen-stimulated lymphocytes. As described above, corresponding MSCs were previously plated in 24-well plates at 100,000 cells per well in duplicate and prepared for each condition (MSC-naïve, MSC-primed, MSC-chondro).

CFSE-labelled lymphocytes from autologous, MHC-matched and mismatched horses were seeded at 1 × 10^6^ PBL per well (1:10 ratio MSC:PBL), based on previous studies [34,37] with PBL medium containing mitogen (PHA 10 μg/mL) [32,38], using the combinations explained above under experimental design.

Appropriate controls were set along with experimental conditions in duplicate: 500,000 unlabelled (background control) and CFSE-labelled (with and without PHA, non-proliferating and proliferating controls, respectively) were seeded alone in a 96-well U-bottom. All the cultures were maintained for 3 days, after which corresponding analysis were performed, as will be detailed below.

#### 2.4.5. Modified One-Way MLR

To assess the ability of MSCs to stimulate a proliferative response in lymphocytes, modified one-way MLRs were performed. Stimulator MSCs were previously plated at 20,000 cells per well on 24-well plates in duplicate for each condition, as described above. Autologous, MHC-matched and mismatched responder CFSE-stained PBLs were seeded at 1 × 10^6^ PBL per well following the experimental design aforementioned, thus resulting in a MSC:PBL ratio of 1:50 [7,34]. Appropriate controls were set as described for immunosuppression assays (unlabelled PBLs for background signal and CFSE-stained PBLs, with and without PHA, as non-proliferating and proliferating internal controls). In addition, classic MLRs were established for each responder. Briefly, MHC-matched and mismatched PBLs were used as stimulators by treating them with 50 μg/mL mitomycin C (Sigma-Aldrich) (37 °C 30′ incubation followed by 2 washes with PBS) to inhibit proliferation [10,39]. Stimulator PBLs were plated at 500,000 PBLs/well in 96-well plates immediately before the addition of 500,000 CFSE-stained responder PBLs (ratio 1:1) to create MHC-matched and mismatched MLRs for all the PBLs tested with MSCs.

All the cultures were maintained for 5 days without media exchange, after which corresponding analyses were performed, as will be detailed below.

### 2.5. Analysis of Lymphocyte Proliferation and Subpopulations

After co-culture, experimental PBLs from proliferation assays (both immunosuppression and modified one-way MLRs) were collected from 24-well plates, centrifuged at 310× *g* 5′, resuspended in PBS, and split for the 2 flow multi-colour panels. The supernatants were collected and centrifuged at 500× *g* 15′ to remove any contaminating cell, and subsequently frozen at –20 °C for further ELISA analysis. Control PBLs in 96-well plates were centrifuged at 310× *g* 5′, supernatants collected as aforementioned, and cells suspended in PBS. Experimental PBLs were then transferred to the 96-well plates along with controls for antibody staining for flow cytometry.

Two multi-colour panels of markers were designed to allow assessment of different lymphocyte subpopulations, along with the proliferation (CFSE dilution). In panel 1, anti-CD3, Pan Ig and CD21 were used to assess T and B cells. In panel 2, anti-CD8, CD4 and CD25 antibodies were used to assess cytotoxic, helper and regulatory T cells. All primary antibodies were selected based on previous reports [40,41,42,43], and used directly conjugated or in combination with appropriate secondary antibodies [44]. All the antibodies were previously titrated in order to determine the optimal dilution. Information regarding antibodies characteristics, dilution and conjugated fluorochromes is presented in Supplementary Materials S2: Tables S2.1 and S2.2.

For panel 1 staining, all control and experimental PBLs were blocked with 100 µL/well of 10% normal goat serum (Panbiotech, IBIAN, Zaragoza, Spain) in PBS, washed twice (wash buffer consisting of PBS with 3% FBS and 310× *g* 5′ spinning) and stained with primary anti-Pan Ig-cells antibody. Then, PBLs were washed twice, incubated with corresponding secondary antibody, and washed again prior to staining with anti-CD21 antibody. At this point, viability staining was performed with Ghost dye Violet 450 (Tonbo Biosciences, Bio-Rad, Barcelona, Spain) as per manufacturer’s instructions and prior to fixation and permeabilization. Cells were washed twice in PBS and fixation/permeabilization were performed using Leucoperm reagents (Bio-Rad, Barcelona, Spain) according to manufacturer’s indications, provided that the anti-CD3 antibody was directed against an intra-cellular epitope. Immediately after the permeabilization step, PBLs were blocked with 100 µL/well of 10% normal mouse serum in PBS, washed and stained with primary anti-CD3 antibody. After two washing steps, cells were incubated with corresponding secondary antibody. Cells were washed twice and fixed with 4% paraformaldehyde (Fisher Scientific) in PBS for 15′ 4 °C, with one washing step afterwards.

For panel 2, all PBLs were blocked in PBS containing 10% donkey serum and 10% rat serum (100 µL/well) (both from Panbiotech). After two washing steps, cells were stained simultaneously with both primary anti-CD4 and anti-CD25 antibodies, washed twice, and incubated with corresponding secondary antibodies. After two washing steps, cells were stained with primary anti-CD8 antibody. Subsequently, cells were washed twice with PBS and incubated with viability dye prior to fixing them, as described above.

All blocking steps and incubation with primary antibodies were performed at 4 °C for 30′. All the incubations with secondary antibodies were carried out at 4 °C for 20′. The volume per well of each primary or secondary antibody was of 50 µL. Two technical replicates per sample were performed and all the procedures were carried out protecting the samples from light. All samples in each panel were subjected to the same steps and were kept in 3% FBS in PBS at 4 °C in the dark up to 24 h for analysis.

Samples were analysed in a Gallios flow cytometer (Beckman Coulter, Madrid, Spain), acquiring a minimum of 10,000 events per sample. Flow cytometry data was analysed with FCS Express 7 Flow software (De Novo Software, Pasadena, CA, USA).

Unstained and secondary controls (cells stained with secondary antibodies alone) were used to assess fluorescence background and establish gates. Single colour stains were used for compensation controls and fluorescence minus one (FMO) controls were performed to assess the fluorescence spread from other channels. The population of lymphocytes was first gated in the forward and side scatter (FSC × SSC) plot, and doublets were excluded. Dead cells were subsequently excluded by incorporation of the viability staining (FL9 channel). In panel 1, live cells were gated for T cell population as CD3+ and for B cells as CD3−/pan-Ig +/CD21+ [45]. In panel 2, live cells were gated as cytotoxic and helper T cells as CD8+/CD4− and CD4+/CD8−, respectively. The subpopulation of regulatory T cells (Treg) was gated from the CD4+ cells as CD4+/CD25^high^ [46]. Due to the variability among individuals, for each animal, the percentage of each lymphocyte subpopulation was normalised over the positive control (PHA-stimulated PBLs) in the immunosuppressive assays, and over the MLR-matched control in the one-way MLR assays.

In both immunosuppressive and one-way MLR assays, proliferation was assessed for each lymphocyte subset by studying CFSE dilution (FL1 channel) in cells gated as aforementioned. Unstimulated and unstained PBLs were used to define the autofluorescence (background). Unstimulated and CFSE-labelled PBLs were used to establish the non-proliferating population, considering cells to the left (lower fluorescence intensity) as proliferating. Supplementary Material S2: Figure S2.1 represents the gating strategy.

In the immunosuppressive assays, PHA-stimulated PBLs alone from each horse were used as the positive control and their proliferation was considered as 100%. Lymphocyte proliferation after co-culture was calculated by comparing samples to paired positive control to account for inter-individual variability in PHA response.

In the one-way modified MLRs, PHA-stimulated PBLs served only as internal control to verify their proliferative ability. Proliferation after MSC exposure was normalised over that observed in MLR-matched controls.

### 2.6. Interferon Gamma Secretion Assay

Supernatants collected from both immunosuppression and modified one-way MLR assays were used to evaluate IFNγ production by using a commercially available ELISA kit (Equine IFN-gamma DuoSet ELISA, R&D Systems, REF: DY1586), as previously reported [33,47]. The supernatants from unstimulated and PHA-stimulated PBLs seeded alone were used as negative and positive controls, respectively, for the immunosuppression assays. For the modified one-way MLRs, the supernatants from the classical MLRs with MHC-matched or mismatched PBLs as stimulators were used as the negative and positive control, respectively. All supernatants were diluted 1:1 in reagent diluent.

All the procedures were performed as per manufacturer’s instructions and concentrations determined using a standard curve, including a blank.

The standard curve was set from 31.25 pg/mL to 8000 pg/mL of IFNγ. All the samples and points of the standard curve were run in duplicate. All the colorimetric assays were analysed on a microplate reader (Biotek Synergy HT, Winooski, VA, USA) and read immediately at 450 nm with wavelength correction set to 540 nm. The duplicate readings for each standard, control, and sample were averaged, and the average zero standard optical density was extracted. The standard curve was created generating a four-parameter logistic curve-fit and the concentrations extrapolated were multiplied by the dilution factor.

### 2.7. Statistical Analysis

Statistical analysis was performed with GraphPad Prism 5 software (San Diego, CA, USA). Data sets were checked for normality using the Shapiro–Wilk test, and parametric or non-parametric tests were chosen accordingly. Differences in the surface expression of MHC-I and MHC-II between MSC-naïve and MSC-primed were analysed by paired *t*-test. Results from flow cytometry and ELISA were separately analysed for each assay (immunomodulation or immunogenicity). For each one, data were compared among MSC-naïve, MSC-primed and MSC-chondro, and regarding corresponding controls, in each type of co-culture (autologous, allogeneic MHC-matched or allogeneic MHC-mismatched) by using one-way ANOVA or the Kruskall–Wallis test followed by Dunn’s post-hoc test. The effect of the type of co-culture was analysed by comparing the results for each type of combination (autologous, MHC-matched, MHC-mismatched) for each type of MSC (MSC-naïve, MSC-primed, MSC-chondro) using paired tests (repeated measures ANOVA or Friedman test, followed by Dunn’s post-hoc test). Significance was set as *p* < 0.05 in all cases.

## 3. Results

### 3.1. Surface Expression of MHC-I and MHC-II

**Figure 2:**
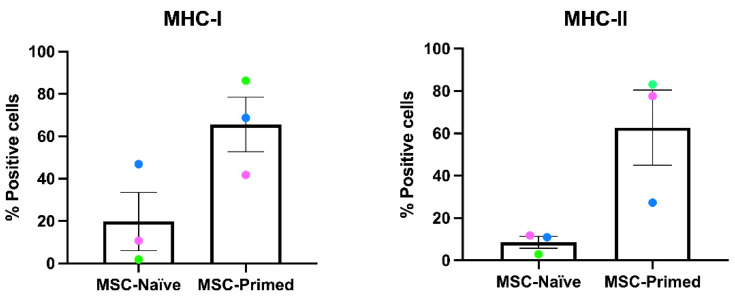
Mean ± S.E.M (*n* = 3) of the percentage of MSC before (MSC-naïve) and after being exposed to proinflammatory cytokines (MSC-primed) positive for the surface markers MHC type I and MHC-II determined by flow cytometry. Each colour dot represents the value for each donor (D1, green dot; D2, pink dot; D3, blue dot). These results corroborated the increased expression of both MHC type I and II in MSC-primed, and the inter-individual variability for MHC expression.

The expression of MHC-I was highly variable among MSCs from the three horses, both before and after priming MSCs with proinflammatory cytokines. The mean value of MHC-I expression was 19% of positive cells for MSC-naïve and 65% for MSC-primed. Expression of MHC-II in MSC-naïve was low and similar among donors (mean of 8.5% positive cells) and experienced a similar increase in the MSCs from the three horses after priming (mean 62%). It is worth noting that MSCs from one donor (D1, green dot) showed very low expression of both MHC-I and MHC-II before priming (MSC-naïve), but these cells experienced the greatest MHC-I and II increase after priming (MSC-primed) (Figure 2).

### 3.2. Changes in Lymphocyte Subpopulations and Proliferation in Immunomodulatory Assays

#### 3.2.1. CD3+ T Lymphocytes

**Figure 3:**
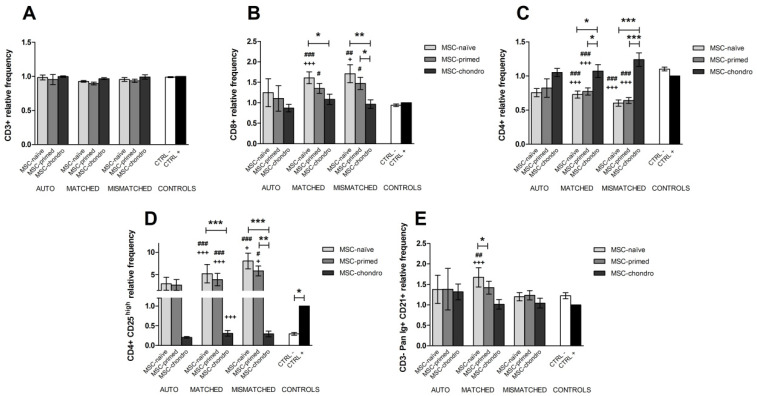
Mean ± SEM of the relative frequency of different lymphocyte subsets in the immunomodulatory assays: (**A**) CD3+ T lymphocytes, (**B**) CD8+ cytotoxic T cells, (**C**) CD4+ helper T cells, (**D**) CD4+ CD25^high^ regulatory T cells, (**E**) CD3−Pan-Ig+ CD21+ B cells. Phytohemagglutinin (PHA)-activated PBLs were exposed in vitro to MSC-naïve (light grey bar), MSC-primed (medium grey bar) and MSC-chondro (dark grey bar). Co-cultures of MSCs and PBLs were autologous (*n* = 3) or allogeneic, matched (*n* = 8) or mismatched (*n* = 7) for the MHC. Data from each PBL donor is normalised over the positive control (CTRL+) consisting of PHA-stimulated PBLs alone from the same donor (value 1), to account for inter-individual variability. Significant differences of each condition over the CTRL+ (black bar) are represented by a cross (+) above the corresponding bar (+ = *p* < 0.05; +++ = *p* < 0.001). Significant differences over the negative control (CTRL−; non-activated PBLs alone, white bar) are represented by hashes (#) above the corresponding bar (# = *p* < 0.05; ## = *p* < 0.01; ### = *p* < 0.001). Significant differences between experimental conditions are represented by a squared line with an asterisk (* = *p* < 0.05; ** = *p* < 0.01; *** = *p* < 0.001).

The percentage of CD3+ T lymphocytes remained similar under the different conditions, so neither the type of combination nor the type of MSCs seemed to produce relevant changes in the global T cell population (Figure 3A).

**Figure 4:**
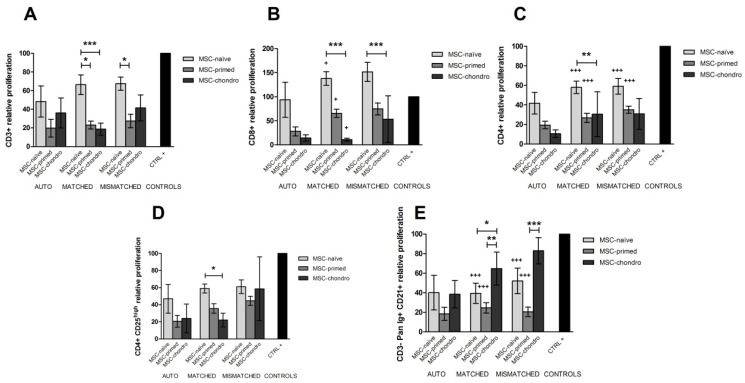
Mean ± SEM of the relative proliferation of different lymphocyte subsets in the immunomodulatory assays: (**A**) CD3+ T lymphocytes, (**B**) CD8+ cytotoxic T cells, (**C**) CD4+ helper T cells, (**D**) CD4+ CD25^high^ regulatory T cells, (**E**) CD3−Pan-Ig+ CD21+ B cells. Phytohemagglutinin (PHA)-activated PBLs were exposed in vitro to MSC-naïve (light grey bar), MSC-primed (medium grey bar) and MSC-chondro (dark grey bar). Co-cultures of MSCs and PBLs were autologous (*n* = 3) or allogeneic, matched (*n* = 8) or mismatched (*n* = 7) for the MHC. Data from each PBL donor is normalised over the positive control (CTRL+) consisting of PHA-stimulated PBLs alone from the same donor (100%, maximum proliferation) to account for inter-individual variability. Significant differences of each condition over the CTRL+ (black bar) are represented by a cross (+) above the corresponding bar (+ = *p* < 0.05; +++ = *p* < 0.001). Significant differences between experimental conditions are represented by a squared line with an asterisk (* = *p* < 0.05; ** = *p* < 0.01; *** = *p* < 0.001).

However, the proliferation of CD3+ T cells was reduced in the presence of all types of MSCs and for all the combinations (autologous and allogeneic MHC-matched and mismatched) compared to PHA-stimulated lymphocytes alone. Overall, MSC-primed were superior suppressing the proliferation of lymphocytes, followed by MSC-chondro and MSC-naïve. In the MHC-matched co-cultures, CD3+ T cell proliferation was significantly reduced by MSC-primed (*p* < 0.05) and by MSC-chondro (*p* < 0.001) compared to MSC-naïve, whereas in MHC-mismatched combination, the suppression was significant only between MSC-primed and MSC-naïve (*p* < 0.05). Significant differences were not observed between MSC types under autologous combinations, likely because of the lower *n*. Nevertheless, autologous MSCs tended to further suppress CD3+ T cell proliferation, followed by allogeneic matched and mismatched MSCs (non-significant) (Figure 4A).

#### 3.2.2. CD8+ Cytotoxic and CD4+ Helper T Lymphocytes

When looking at specific T cell populations, CD8+ cytotoxic and CD4+ helper T cells showed opposite changes. The percentage of CD8+ T cells tended to increase in the presence of MSCs, as shown by significant differences over PBLs alone in both positive and negative controls. Specifically, the population of CD8+ T cells was significantly increased by MSC-naïve MHC-matched (*p* < 0.001) and mismatched (*p* < 0.05) compared to PHA-stimulated PBLs alone. Compared to the negative control (non-stimulated PBLs alone), matched MSC-naïve (*p* < 0.001), mismatched MSC-naïve (*p* < 0.05), matched MSC-primed (*p* < 0.01) and mismatched MSC-primed (*p* < 0.05) significantly raised the CD8+ population. There were not significant differences among combinations, but a tendency was observed with MHC-mismatched MSCs inducing higher CD8+ percentages, followed by MHC-matched and autologous MSCs. Overall, the type of MSCs (naïve, primed or chondro) also influenced the T cytotoxic population, with MSC-naïve promoting higher percentages of CD8+ cells, followed by MSC-primed and MSC-chondro. Specifically, this increase was statistically significant between MSC-naïve and MSC-chondro for both MHC-matched (*p* < 0.05) and MHC-mismatched (*p* < 0.01) combinations. In the mismatched setting, MSC-primed also induced a significantly higher percentage of CD8+ T cells compared to MSC-chondro (*p* < 0.05) (Figure 3B).

On the contrary, the population of CD4+ T cells tended to decrease in the presence of MSCs. The percentage of helper T cells was significantly reduced over both the positive and negative controls when exposed to MHC-matched or MHC-mismatched MSC-naïve and MSC-primed (*p* < 0.001 in all cases). In spite of no significant differences detected among combinations, a tendency was observed with autologous MSCs inducing higher CD4+ percentages, followed by MHC-matched and mismatched MSCs—on the contrary to that observed for CD8+ T cells. In addition, inversely to the cytotoxic T cell population, MSC-naïve promoted lower percentages of CD4+ cells, followed by MSC-primed and MSC-chondro. Specifically, PBLs co-cultured with MSC-naïve and MSC-primed showed lower percentages of CD4+ cells than those exposed to MSC-chondro in both MHC-matched (*p* < 0.05) and mismatched (*p* < 0.001) allogeneic settings (Figure 3C).

Regarding the proliferation of these subpopulations, MSC-primed and MSC-chondro tended to suppress proliferating CD8+ T cytotoxic cells over the positive control, but MSC-naïve promoted an increase in the proliferation of CD8+ cells. These differences were statistically significant only in the MHC-matched setting (*p* < 0.05 for all the three types of MSCs). Interestingly, MSC-chondro showed the highest suppressive capacity, followed by MSC-primed. Specifically, MSC-chondro significantly suppressed CD8+ T cells proliferation compared to MSC-naïve in both MHC-matched and mismatched co-cultures (*p* < 0.001). Significant differences were not observed between MSC types under autologous combinations, as reported for CD3+ T cells. Significant differences were neither seen among combinations, but a tendency towards further suppressive capacity of autologous MSCs over allogeneic ones was noted, as well as the aforementioned for CD3+ lymphocytes (Figure 4B).

Proliferation of CD4+ T cells was suppressed in all the co-cultures with MSCs. Compared to PHA-stimulated PBLs alone, MSC-naïve and MSC-primed significantly reduced CD4+ cells proliferation in both MHC-matched and mismatched combinations (*p* < 0.001 for all conditions). MSC-chondro also showed suppressive capacity of CD4+ cells, but it was more variable, being significant only over MSC-naïve in the MHC-matched setting (*p* < 0.01). Similarly to that described for CD3+ and CD8+ cells, the effect of the type of combination was not statistically significant, but autologous MSCs tended to show further suppression (Figure 4C).

#### 3.2.3. CD4+ CD25^high^ Regulatory T Cells

In this study, Treg were gated as CD4+ CD25^high^ cells. Phytohemagglutinin increased the percentage of CD4+ CD25^high^, as shown in the positive control over the negative one (*p* < 0.05), and as also previously reported using a similar lectin mitogen [48]. In the presence of MSC-naïve and MSC-primed, PHA-activated PBLs further increased the percentage of CD4+ CD25^high^ T cells, whereas MSC-chondro clearly tended to decrease this population. Specifically, and compared to the positive control, Treg population increased when co-cultured with MSC-naïve and MSC-primed, either MHC-matched (*p* < 0.001) or MHC-mismatched (*p* < 0.05), and decreased when exposed to MHC-matched MSC-chondro (*p* < 0.001). Similar increases were also noted over the negative control (MHC-matched MSC-naïve and primed, *p* < 0.001; MHC-mismatched MSC-naïve, *p* < 0.001; MHC-mismatched MSC-primed, *p* < 0.05). The effect of the combination followed a clear tendency in spite of no significant differences observed, with MHC-mismatched MSCs inducing higher levels of Treg cells, followed by MHC-matched and autologous MSCs. The effect of the type of MSCs was also clear, with MSC-naïve further increasing Treg cells, followed by MSC-primed. In particular, MHC-matched and mismatched MSC-naïve promoted higher percentages of Tregs over MSC-chondro (*p* < 0.001 in both cases), and MHC-mismatched MSC-primed also increased this population compared to MSC-chondro (*p* < 0.01) (Figure 3D).

Despite of the fact that MSC-naïve and MSC-primed can increase the CD4+ CD25^high^ population, all the co-cultures suppressed the proliferation of these cells compared to the positive control, but this reduction was not statistically significant in any condition. Suppression of Treg proliferation was stronger for MHC-matched MSC-chondro over MSC-naïve (*p* < 0.05), which may be related to the tendency of MSC-chondro to reduce the percentage of these cells. No clear effect of the type of combination was observed, but overall autologous MSCs appeared to be more suppressive (Figure 4D).

#### 3.2.4. CD3− Pan-Ig+ CD21+ B Cells

B cells were gated as CD3− Pan-Ig+ CD21+ and this population tended to increase in the presence of MSCs, especially with MSC-naïve. Specifically, MHC-matched MSC-naïve promoted higher percentage of CD3− Pan-Ig+ CD21+ compared to the positive (*p* < 0.001) and negative (*p* < 0.01) controls, and also to MSC-primed (*p* < 0.05) (Figure 3E).

When looking at the proliferation of B cells, MSCs had a clear suppressive effect. Compared to PHA-activated PBLs alone (positive control), MHC-matched and mismatched MSC-naïve and MSC-primed significantly suppressed CD3− Pan-Ig+ CD21+ proliferation (*p* < 0.001 in all the four comparisons). When comparing types of MSCs, MSC-primed elicited a higher suppressive potential of this population, followed by MSC-naïve and MSC-chondro. In particular, suppression of the B cell population was further suppressed by MHC-matched MSC-primed (*p* < 0.01) and MSC-naïve (*p* < 0.05), and by MHC-mismatched MSC-primed (*p* < 0.001), compared to corresponding MSC-chondro. The type of combination did not significantly influence the suppression of B cells proliferation, but autologous MSCs tended to further reduce this proliferating population (Figure 4E).

### 3.3. Changes in Lymphocyte Subpopulations and Proliferation in Immunogenicity Assays

#### 3.3.1. CD3+ T Lymphocytes

**Figure 5:**
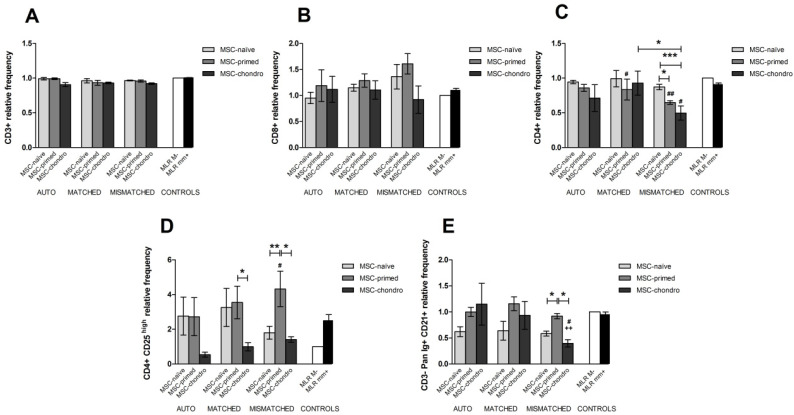
Mean ± SEM of the relative frequency of different lymphocyte subsets in the immunogenicity assays (one-way modified mixed lymphocyte reaction): (**A**) CD3+ T lymphocytes, (**B**) CD8+ cytotoxic T cells, (**C**) CD4+ helper T cells, (**D**) CD4+ CD25^high^ regulatory T cells, (**E**) CD3−Pan-Ig+ CD21+ B cells. Non-activated PBLs were exposed in vitro to MSC-naïve (light grey bar), MSC-primed (medium grey bar) and MSC-chondro (dark grey bar). Co-cultures of MSCs and PBLs were autologous (*n* = 3) or allogeneic, matched (*n* = 8) or mismatched (*n* = 7) for the MHC. Data from each PBL donor is normalised over the negative control (MLR M−, matched MLR) consisting of responder PBLs from the same donor exposed to MHC-matched stimulator PBLs (value 1), to account for inter-individual variability. Significant differences of each condition over the positive control (MLR mm+, mismatched MLR consisting of responder PBLs exposed to MHC-mismatched stimulator PBLs; black bar) are represented by a cross (+) above the corresponding bar (++ = *p* < 0.01). Significant differences over the negative control (MLR M−, white bar) are represented by hashes (#) above the corresponding bar (# = *p* < 0.05; ## = *p* < 0.01). Significant differences between experimental conditions are represented by a squared line with an asterisk (* = *p* < 0.05; ** = *p* < 0.01; *** = *p* < 0.001).

The percentage of CD3+ T cells did not significantly change in response to the different MSCs in the modified one-way MLRs, neither regarding their type (MSC-naïve, MSC-primed, MSC-chondro), nor regarding the combination (autologous, allogeneic MHC-matched or mismatched) (Figure 5A).

**Figure 6:**
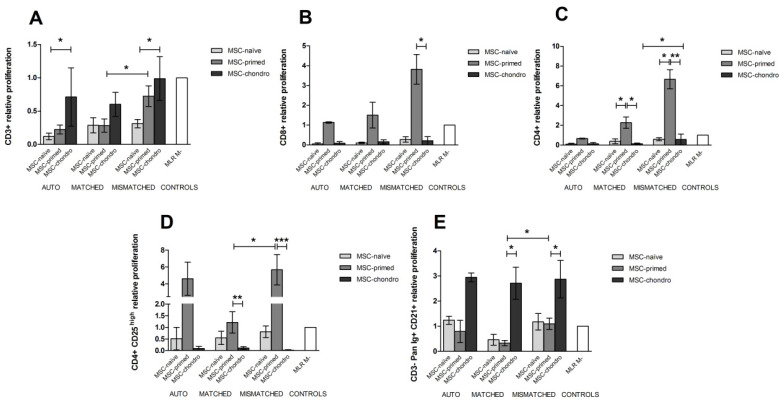
Mean ± SEM of the relative proliferation of different lymphocyte subsets in the immunogenicity assays (one-way modified mixed lymphocyte reaction): (**A**) CD3+ T lymphocytes, (**B**) CD8+ cytotoxic T cells, (**C**) CD4+ helper T cells, (**D**) CD4+ CD25^high^ regulatory T cells, (**E**) CD3−Pan-Ig+ CD21+ B cells. Non-activated PBLs were exposed in vitro to MSC-naïve (light grey bar), MSC-primed (medium grey bar) and MSC-chondro (dark grey bar). Co-cultures of MSCs and PBLs were autologous (*n* = 3) or allogeneic, matched (*n* = 8) or mismatched (*n* = 7) for the MHC. Proliferation of each PBL donor is normalised over the proliferation observed in the negative control (MLR M−, matched MLR) consisting of responder PBLs from the same donor exposed to MHC-matched stimulator PBLs (value 1), to account for inter-individual variability. Significant differences over the negative control (MLR M−, white bar) were not observed. Significant differences between experimental conditions are represented by a squared line with an asterisk (* = *p* < 0.05; ** = *p* < 0.01; *** = *p* < 0.001).

Compared to the negative control (MLR-matched), proliferation of T cells was not significantly induced under any condition. Actually, a lower proliferation of T cells was observed when co-cultured with MSC-naïve and MSC-primed in all the three combinations. Specifically, proliferation of T cells was significantly lower with MSC-naïve than with MSC-chondro in both autologous and MHC-mismatched settings (*p* < 0.05 in both conditions). Regarding the mismatched co-cultures, these tended to produce a higher T cell response, followed by MHC-matched and autologous combinations. Nevertheless, the difference was only significant for MSC-primed between mismatched and matched conditions (*p* < 0.05) (Figure 6A).

#### 3.3.2. CD8+ Cytotoxic and CD4+ Helper T Lymphocytes

The percentage of CD8+ T cells did not significantly change under any condition in the one-way modified MLR assays, contrary to that observed in the immune suppression assays (Figure 5B). However, the percentage of CD4+ T cells was reduced in the presence of MSC-primed in the allogeneic MHC-matched (*p* < 0.05) and mismatched (*p* < 0.01) combinations, and after the exposure to MSC-chondro in the MHC-mismatched scenario (*p* < 0.05), compared to the MLR-matched control. Furthermore, in the mismatched co-cultures, MSC-chondro (*p* < 0.001) and MSC-primed (*p* < 0.05) reduced the percentage of CD4+ T cells compared to the MSC-naïve. Overall, mismatched combinations tended to further reduce the percentage of helper T cells, but only MSC-chondro showed a significant reduction (*p* < 0.05) compared to the matched setting (Figure 5C).

A statistically significant increase of CD8+ T cells proliferation was neither observed compared to the MLR-matched control under any condition, but a marked tendency towards cytotoxic T cells induction was observed with MSC-primed. Actually, CD8+ T cell proliferation was higher in the presence of MSC-primed compared to MSC-naïve and MSC-chondro in the three combinations, but the difference was statistically significant only regarding MSC-chondro in the mismatched setting (*p* < 0.05) (Figure 6B). Proliferation of CD4+ T cells followed a similar pattern to CD8+ T cells: whereas no significant differences were observed between any condition and the MLR-matched control, MSC-primed tended to induce T helper proliferation in the two allogeneic settings. Specifically, proliferation of CD4+ T cells was higher after exposure to MSC-primed compared to MSC-naïve (*p* < 0.05) and to MSC-chondro in both the MHC-matched (*p* < 0.05) and mismatched (*p* < 0.01) scenarios. Significant differences among conditions were only observed for MSC-chondro, with higher proliferation in the MHC-mismatched combination compared to the MHC-matched one, but not exceeding the MLR-matched values (Figure 6C).

#### 3.3.3. CD4+ CD25^high^ Regulatory T Cells

Overall, MSC-naïve and MSC-primed tended to increase the percentage of CD4+ CD25^high^ and MSC-chondro to reduce it, regardless of the type of combination. Specifically, MHC-mismatched MSC-primed significantly increased the CD4+ CD25^high^ population (*p* < 0.05) compared to the MLR-matched control and to both MSC-naïve (*p* < 0.01; MHC-mismatched) and MSC-chondro (*p* < 0.05; MHC-matched and mismatched) (Figure 5D).

Similarly to that observed for cytotoxic and helper T cells, Treg cells were induced to proliferate after exposure to MSC-primed regardless of the type of combination. However, the proliferation observed was not statistically different to that reported for the MLR-matched control. Nevertheless, MSC-primed significantly induced proliferation of Tregs compared to MSC-chondro in both the allogeneic MHC-matched (*p* < 0.01) and mismatched (*p* < 0.001) combinations, as also seen with CD4+ helper T cells. Furthermore, the proliferation induced by MSC-primed was significantly higher in the mismatched setting compared to the matched (*p* < 0.05) (Figure 6D).

#### 3.3.4. CD3− Pan-Ig+ CD21+ B Cells

The frequency of the B cell population did not significantly change compared to control MLRs after exposure to different types of MSCs in autologous and allogeneic MHC-matched combination. Overall, MSC-naïve tended to further diminish the percentage of B cells. However, in the MHC-mismatched combination, MSC-chondro promoted a greater reduction, being significant compared to both MLR-matched (*p* < 0.05) and mismatched (*p* < 0.01) controls. In this setting, both MSC-chondro and MSC-naïve elicited a reduction of B cells compared to MSC-primed (*p* < 0.05 in both cases) (Figure 5E).

In the three combinations, proliferation of B cells was similar to that in the MLR-matched control for MSC-naïve and MSC-primed, whereas it was higher for MSC-chondro but not statistically significant. However, B cell proliferation elicited by MSC-chondro was significantly higher (*p* < 0.05) than that observed with MSC-primed in both allogeneic MHC-matched and mismatched settings. Among combinations, proliferation of B cells exposed to MSC-primed was higher (*p* < 0.05) in the mismatched scenario than in the matched, but did not differ from the MLR-matched control (Figure 6E).

### 3.4. Interferon Gamma (IFNγ) Production in Immunomodulatory and Immunogenicity Assays

**Figure 7:**
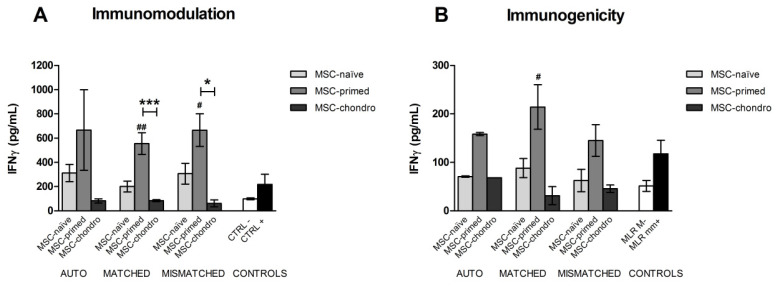
Mean ± SEM concentration (pg/mL) of interferon gamma (IFNɣ) in co-culture supernatants from immunomodulatory (**A**) and immunogenicity (**B**) assays. Phytohemagglutinin (PHA)-activated PBLs (**A**) or non-activated PBLs (**B**) were exposed in vitro to MSC-naïve (light grey bar), MSC-primed (medium grey bar) and MSC-chondro (dark grey bar). Co-cultures of MSCs and PBLs were autologous (*n* = 3) or allogeneic, matched (*n* = 8) or mismatched (*n* = 7) for the MHC. Significant differences of each condition over the positive controls were not observed. Significant differences over the negative controls are represented by hashes (#) above the corresponding bar (# = *p* < 0.05; ## = *p* < 0.01). Significant differences between experimental conditions are represented by squared line with asterisk (* = *p* < 0.05; *** = *p* < 0.001).

The concentration of IFNγ was measured in co-culture supernatants from both immunomodulatory and immunogenicity assays as reflection of lymphocyte activation. Values of IFNγ were overall higher in the immunomodulatory assays, where PBLs were activated by PHA. Significant differences among autologous, allogeneic MHC-matched and MHC-mismatched co-cultures were not observed, neither in immunomodulatory assays nor in the immunogenicity ones, but for both types of assays, IFNγ concentration was higher in the co-cultures with MSC-primed (Figure 7).

In the immunomodulatory assay, co-cultures with MSC-naïve showed IFNγ concentrations similar or slightly higher to the positive control (PHA-stimulated PBLs alone), but significant differences were not observed. IFNγ presence in co-cultures with MSC-chondro was similar to that in the negative control (unstimulated PBLs alone). However, supernatant from MSC-primed co-cultures contained significantly higher concentrations of IFNγ than the negative control in both MHC-matched (*p* < 0.01) and MHC-mismatched (*p* < 0.05) combinations. In addition, IFNγ in these conditions also increased significantly compared to corresponding MSC-chondro co-cultures (*p* < 0.001 for MHC-matched, *p* < 0.05 for MHC-mismatched) (Figure 7A).

In the one-way MLRs (immunogenicity assays), both MSC-naïve and MSC-chondro induced IFNγ secretion in amounts similar to the negative control (MLR-matched) and lower than in the positive control (MLR-mismatched), but no significant differences over controls were observed. Presence of IFNγ in the supernatants from MSC-primed co-cultures was higher than with the other MSC types and compared to controls, but significant differences were only observed between the MHC-matched MSC-primed co-culture and the negative control (*p* < 0.05) (Figure 7B).

## 4. Discussion

To the best of authors’ knowledge, this is the first study in the equine species assessing the effect of proinflammatory priming, chondrogenic differentiation, and MHC compatibility on the immunomodulatory and immunogenic potentials of MSCs. Under the conditions of this study, equine MSCs stimulated by proinflammatory cytokines (MSC-primed) were more immunosuppressive and presented mild immunogenicity compared to non-manipulated MSCs (MSC-naïve). Interestingly, equine MSCs differentiated into chondrocytes (MSC-chondro) did not lose their regulatory capacity, and neither did they significantly increase their immunogenicity. In addition, autologous MSCs were slightly more suppressive, whereas allogeneic MHC-mismatched MSCs tended to be more immunogenic, especially when they were primed with cytokines.

Previous studies have shown that both autologous and allogenic MSCs significantly suppressed the proliferation of PHA-activated T cells [32,39,49], but fewer studies have analysed the ability of MSCs to stimulate an immune response in resting lymphocytes [7,34,50]. In addition, the effects of MSCs under different conditions on specific lymphocyte subpopulations, and the way these populations change and proliferate after co-culture in vitro, has been barely studied in the horse. Overall, and in agreement with previous reports, co-culture assays conducted in this study showed that allogeneic MSCs are able to suppress activated lymphocytes. Moreover, allogeneic MSCs do not stimulate marked changes on the proliferation and frequency of different lymphocyte subpopulations.

According to our initial hypothesis, MSC-primed were more immune suppressive but also more immunogenic than MSC-naïve. However, we also hypothesized that MSC-chondro would lose their regulatory ability and would be more easily recognized by the immune cells; however, it turned out that differentiated MSCs were able to suppress the proliferation of different lymphocyte subpopulations in a similar way to MSC-primed, and did not provoke a marked proliferative response in resting lymphocytes. Therefore, the capacity of suppressing CD3+ T cells was enhanced in both MSC-primed and MSC-chondro over MSC-naïve. Actually, when analysing the two main subpopulations of T cells, MSC-naïve were not able to suppress the proliferation of CD8+ cytotoxic T cells, indicating that the suppression in T cell proliferation elicited by these cells was exclusively due to the suppression of CD4+ helper T cells. Thus, and according to previous studies in the horse [34,51], a suppressive effect of MSC-naïve is observed in the general T cell population. However, contrary to our findings, Ranera et al. (2016) found that untreated MSCs were able to suppress the proliferation of CD8+ lymphocytes, while CD4+ cells were only slightly modified [32]. Meanwhile, both MSC-primed and MSC-chondro decreased the proliferation of both cytotoxic and helper T cells in a similar way, suggesting a further activation of their immunomodulatory properties over untreated MSCs, since cytotoxic T cells (CD8+) are primarily involved in the destruction of cells presenting foreign antigens, and T helper cells (CD4+) play an important role in establishing and maximizing the immune response [52].

Even though MSC-primed and MSC-chondro similarly suppressed the proliferation of cytotoxic and helper T cells, they differ in their behaviour regarding Treg and B cells. An increase in Tregs would be related to the immunosuppressive ability of MSCs, as these lymphocytes would help dampen the adaptive immune response and prevent rejection of foreign cells by the host [53]. In the one-way MLRs conducted in our study, relative frequency and proliferation of Treg cells were consistently and significantly increased after exposure to MSC-primed compared to MSC-chondro. In addition, MSC-naïve also induced an increase of Treg relative frequency in the three combinations, similarly to that reported by Kamm et al. (2021), which also showed an increase in Treg lymphocytes in contact with MSCs from universal blood donors and with low MHC class II expression [50]. Furthermore, for the population of B lymphocytes, which principal function is the production of antibodies against foreign antigens [52], MSC-primed showed the most marked suppressive capacity. In agreement with our results, a previous study in human MSCs [54] observed that MSC-primed inhibited the proliferation of B cells over MSCs-naïve. Inversely, for lymphocytes B, MSC-chondro were significantly less suppressive than MSC-primed.

Previous studies measuring the ability of proinflammatory primed equine MSCs to suppress the proliferation of allogeneic activated T cells have shown similar results. The study of Caffi et al. (2020) analysed the effect of pre-conditioning MSC with proinflammatory cytokines (TNFα and IFNγ) and, according to our results, reported that priming MSCs increases their inhibitory effect on lymphocyte proliferation [55]. Another study [51] concluded that MSCs primed with either polyinosinic:polycytidylic acid, lipopolysaccharide, or inflammatory macrophages, produced a similar enhancement in their ability to modulate T cell proliferation. This suppressive ability of MSC-primed would be related to paracrine immunomodulation mechanisms, which are induced by inflammation as reflected by the increase of the expression and secretion of different mediators, such as cyclooxygenase 2 (COX-2), indoleamine 2-3-dioxygenase (IDO), and interleukin 6 (IL-6) [15].

Furthermore, previous studies in other species reported that chondrogenically differentiated rat MSCs had decreased immunomodulatory potential, in disagreement with our results [16]. However, there are also reports on human chondrogenically-induced MSCs showing a regulatory profile similar to undifferentiated cells [56]. Therefore, variability among species could be expected, and thus it is important to explore this scenario in the horse. Beyond the potential inter-species variability, other possible explanations for these findings should be considered. First, the ratio between MSC-chondro and PBLs could have varied over MSC-naïve and MSC-chondro in this experiment. For MSCs-naïve and MSC-primed, the number of cells seeded to reach the appropriate ratio of MSC:PBLs can be controlled, and was chosen based on previous studies [7,32,34]. However, for MSC-chondro, because two weeks were necessary for inducing their differentiation, it is difficult to adjust the exact final number of cells. There is no consensus regarding whether MSCs proliferate or not while differentiating into chondrocytes. Some authors [57] described that proliferation of MSCs in micromasses is decreased during the first week of chondrogenic differentiation, whereas other authors [58] reported that MSCs proliferate as they differentiate into cartilage. In this study and prior to any experimental assay, MSCs were differentiated into chondrocytes by different methods (monolayer, micromass and pellet culture). After two weeks, the extracellular matrix of chondrogenically differentiated MSCs were digested by collagenases, and the number of cells evaluated as previously described [59,60]. Based on these preliminary assays, the micromass technique was chosen as the most consistent method for differentiation under our conditions, but the effect of this variable on the final MSC:PBL ratio cannot be totally disregarded. In addition, the possible presence of undifferentiated MSCs in the MSC-chondro co-cultures could have contributed to the suppression of lymphocyte proliferation observed.

Regarding immunogenicity, and according to our initial hypothesis, MSC-primed induced a proliferative response in cytotoxic and helper T cells. However, B cells were not activated and Treg were increased. However, even though we hypothesized that MSC-chondro would be more immunogenic, they did not increase proliferation or induce changes in the cytotoxic and helper T cell populations. However, MSC-chondro induced proliferation of B cells and showed the lowest ability to stimulate Tregs. Kamm et al. (2021) showed that proliferation of B cells was not stimulated when faced with allogeneic MSCs [50], similarly to that observed with both MHC-matched and mismatched MSC-naïve and MSC-primed in this study. Regarding MSC-chondro, a previous study in rat MSCs [16] reported a significant increase in B cells when co-cultured with chondrogenically differentiated cells, similarly to our findings.

The increased immunogenicity of MSC-primed would be associated with the increase in MHC expression. The upregulation of MHC-I and MHC-II after priming MSCs observed in this study agrees with previous reports in the equine species [7,15]. A recent study in horses [50] described that the MHC expression level in MSCs largely depends on the horse donor, and these cells could be classified as MHC class II-high or low expression, which would affect their immune recognition. According to our results, the MSCs in our study would be classified as MHC-II low, but will change into the MHC-II high category after cytokine priming, with a parallel increase in their capacity to stimulate an immune response.

Studies reporting immune response against MSC-chondro have also shown increased levels of MHC surface expression compared to undifferentiated MSCs [16]. In a previous work from our group, an upregulation of MHC-I and II was observed after chondrogenic differentiation of equine MSCs [17]. However, in the current study, immunogenicity of MSC-chondro was not observed, except for the B cells response. In the equine species, there is only one study assessing the capacity of chondrogenic induced equine allogeneic peripheral blood-derived MSCs (ciMSCs) to activate lymphocytes [18]. Their results showed no cellular immune response in one-way MLRs with ciMSCs, and no immunomodulatory capacity of these cells in immunosuppressive assays either; although, it should be noted that all co-cultures were carried out from 5 days to one year and a half after treating 10 horses with ciMSCs. In addition, in their study, the MHC-haplotype of ciMSC donors and recipients was not established, and neither were lymphocyte subpopulations assessed. It should also be considered that the methodology for chondrogenic induction varies between this and our study, which might explain the lack of immunomodulatory properties seen by Van Hecke et al. (2021) [18].

In our study, MSC-chondro were thoroughly washed prior to adding PBLs but the possible effect of TGF-β3 in the differentiation media regarding their immunogenic profile should also be noted, provided that TGF-β2 can diminish MHC expression in equine MSCs [61]. These authors observed that TGF-β2-treated MHC-mismatched MSCs, which have reduced MHC-I surface expression, and induced less T cell receptor downregulation and cytotoxicity than untreated MHC-mismatched MSCs in vitro, thus suggesting that treatment with this growth factor could reduce the cell-mediated immunogenicity of MHC-mismatched MSCs in vitro.

We also hypothesized that MHC-matched MSCs would generate similar results than autologous ones, while MHC-mismatched MSCs would present similar regulatory capacity but increased immunogenicity. Even though we observed similar results in both immunomodulatory and immunogenicity assays for the three types of combinations, it should be noted that the autologous group tended to further supress the proliferation of all lymphocyte subpopulations, followed by the allogeneic MHC-matched group and the MHC-mismatched one. Furthermore, MHC-mismatched MSCs tended to induce higher lymphocyte proliferation, but this effect was only obvious with MSC-primed.

Ranera et al. (2016) studied the immunomodulatory ability of MSCs co-cultured with activated PBLs of autologous and MHC-mismatched horses, and showed that both of them exhibited a similar capacity to significantly reduce lymphocyte proliferation in vitro [32]. However, in this study MHC-matched co-cultures were not analysed and only MSC-naïve were tested. Another study [34] showed that both autologous and allogeneic MSCs decreased proliferation of activated lymphocytes. Both autologous and allogeneic MSCs appeared to be equally immunosuppressive, and no difference was noted between them, but only CD3+ T cells were analysed. For the general T cell population, our study also showed similar results between combinations for MSCs-naïve, but further tendencies were observed when assessing lymphocyte subpopulations and MSCs under different conditions. Moreover, in the study of Colbath et al. (2017), MHC haplotypes of horses were not established and they reported a low expression of MHC-II in MSCs, which remained low after the co-cultures [34].

To assess the ability of MSCs to activate lymphocytes, Colbath et al. (2017) also co-cultured autologous or allogenic MSCs with inactivated PBLs, and concluded that equine allogeneic MSCs were not inherently more immunogenic in terms of T cell activation, as they only induced a mild lymphocyte proliferation in vitro, similar to that observed with autologous MSCs [34]. In our study, a higher immunogenic response in cytotoxic and helper T lymphocytes were observed in MHC-mismatched co-cultures when MSCs were primed, in agreement with [50], which observed increased proliferation of CD8+ and CD4+ T cells after co-culture with MHC-mismatched MSCs showing MHC-II high expression. Nevertheless, Kamm et al. (2021) concluded that differences between autologous and allogeneic MSCs were minimal regarding their effect on the activation of lymphocytes [50]. On the contrary, another in vitro study where MHC-haplotypes were controlled showed that MHC-mismatched MSCs induced lymphocyte activation [7]. Their results were in agreement with our findings, as Schnabel et al. (2014) revealed that MHC-mismatched MHC class II-positive MSCs caused a significant increase in the proliferation of T cells compared to MHC-matched MSCs and to MHC-mismatched MHC class II-negative, concluding that MHC-mismatched MSCs induced greater lymphocyte activation in vitro, particularly when MHC-II expression was high, as it was observed with MSC-primed in our study [7].

It should also be considered that, in the autologous group of our study, only three horses were involved. This *n* was lower than in the allogeneic groups and could help explain why in most autologous conditions no significant differences appeared among MSC types, even though they followed the same trend as allogeneic MHC-matched and mismatched groups. However, this setup was chosen to use the same MSCs across all the experiments, avoiding the involvement of more animals and the increase of inter-individual variability.

Finally, IFNγ was determined in co-culture supernatants as this proinflammatory cytokine is related to cell-mediated immunity produced by T lymphocytes. The principal function of IFNγ is to stimulate both innate and adaptive immune responses [62], so its secretion indicates expansion of CD8+ or CD4+ effector and memory cells against donor MSCs [5]. However, other authors report that IFNγ production stimulates the regulatory ability of MSCs, thus subsequently reducing the proliferation of immune cells [63]. In our study, overall lower secretion of IFNγ was observed in the immunogenicity assays compared to the immunomodulatory ones, but it must be considered that in the latter, the lymphocytes were mitogen-activated and thus produce higher concentrations of IFNγ [34,47,49]. In both immunomodulatory and immunogenicity assays of our study, lymphocytes co-cultured with MSC-chondro showed the lowest IFNγ secretion, and lymphocytes co-cultured with MSC-primed resulted in the highest secretion.

In the immunomodulatory assays, and contrary to that previously reported [39,49], a significant reduction in IFNγ secretion over the positive control was not observed after co-culture of activated lymphocytes with the different types of MSCs and in the different combinations. Actually, IFNγ secretion increased in the presence of MSC-primed in both immunomodulatory and immunogenicity co-cultures. This finding could reflect a further activation of lymphocytes in the presence of MSC-primed, as seen by increased proliferation of CD8+ and CD4+ T cells in the one-way MLRs of this study and, according to their increased MHC expression, also observed in previous studies [55]. However, the possible presence of residual exogenous IFNγ from the priming process prior to co-culturing MSCs with lymphocytes should also be considered, even though cells were washed with PBS.

At the same time, MSC-naïve and MSC-chondro did not significantly reduce IFNγ secretion by activated lymphocytes, but neither stimulated their secretion over the controls in resting ones. Secretion of IFNγ by lymphocytes in the presence MSC-chondro agrees with observations discussed above regarding suppression of CD8+ and CD4+ T cell proliferation in immunomodulatory assays and lack of their induction in one-way MLRs, further pointing at the ability of equine MSCs to maintain their regulatory properties and not increase their immunogenicity after chondrogenic differentiation.

Finally, and in addition to the limitations discussed along this section concerning the lower *n* of the autologous group, the challenges of MSC-chondro co-cultures and possible interferences of molecules in the culture media, few other points should also be noted. First, for one of the MSC donors (D2), it was only possible to find two other MHC-matched horses (A1, A2). The heterogeneity reported in ELA haplotypes [20,21] likely prevented finding a third matched animal to complete the group, even after a large screening. Nevertheless, the *n* of this study is similar to that in related reports [6,35], since the complexity of randomly finding compatible animals makes working with larger study groups difficult. Secondly, MHC haplotypes were determined as previously described but, since familiar information of horses was not available, only homozygous animals could be assigned as definitive [20,21]. Third, using mitogens such as PHA is a simple and effective method for stimulating PBLs in immune suppression assays, and has been widely reported [39,47], but does not completely resemble the in vivo activation. It should also be considered that in vitro assays provide valuable information to guide in vivo research, but the complexity of the interactions with the immune system cannot be completely reproduced.

## 5. Conclusions

This study reports how inflammation, differentiation, and compatibility for the MHC can influence the immunological properties of equine MSCs. Priming MSCs with proinflammatory cytokines activates their regulatory potential, as seen by decreased proliferation of cytotoxic and helper T cells, and B cells. However, inflammation can also increase the immune recognition of these cells through induction of MHC expression, thus making the allogeneic MHC-mismatched MSCs more likely to be targeted by the immune system. Importantly, equine MSCs do not lose their regulatory ability, and neither increase their immunogenicity after chondrogenic differentiation, but have reduced capacity to stimulate Treg cells and can stimulate the proliferation of B cells. Even though lymphocyte proliferation assays are important tools to assess both the immunomodulatory and immune evasive properties of MSCs, and thus exploring their therapeutic potential and immunogenicity, in vivo studies are needed to fully comprehend the complexity of the interactions of MSCs with the recipient immune system to develop safe and effective cell therapies.

## Figures and Tables

**Table 1:** List of major histocompatibility complex (MHC) microsatellite haplotypes identified in the horses enrolled in the study.

	MHC Class I		MHC Class III		MHC Class II						
Microsatellite Loci	UMNJH-38	COR110	ABGe9019	UMNe65	ABGe9030	EQMHC 1	COR112	COR113	UM011	COR114	
Horses ID											Haplotype
**D1**	165	221	301	261	215	190	262	270	179	241	HapPRE10
	165	221	301	261	215	190	262	270	179	241	HapPRE10
R1	165	221	301	261	215	190	262	270	179	241	HapPRE10
	156	215	301	261	215	190	262	270	179	241	HapPRE10-like
R2	165	221	301	261	215	190	262	270	179	241	HapPRE10
	156	221	320	250	219	190	254	270	172	249	HapMAI06
R3	165	221	301	261	215	190	262	270	179	241	HapPRE10
	156	207	318	263	215	184	262	260	172	243	HapPRE31
**D2**	156	217	312	261	205	194	258	260	169	243	HapMAI04
	156	217	312	261	205	194	258	260	169	243	HapMAI04
A1	156	217	312	261	205	194	258	260	169	243	HapMAI04
	156	205	305	253	205	194	266	268	174	234	HapPRE01
A2	156	217	312	261	205	194	258	260	169	243	HapMAI04
	156	207	312	263	211	192	264	270	172	249	Unknown1
**D3**	156	221	314	259	215	190	262	272	169	255	HapPRE11
	156	221	314	259	215	190	262	272	169	255	HapPRE11
C1	156	221	314	259	215	190	262	272	169	255	HapPRE11
	156	221	312	261	205	190	262	270	180	245	Unknown2
C2	156	221	314	259	215	190	262	272	169	255	HapPRE11
	156	211	301	259	209	192	262	268	174	234	A2 *
C3	156	221	314	259	215	190	262	272	169	255	HapPRE11
	156	207	314	261	215	190	262	270	180	247	HapPRE26

MHC homozygous horses are indicated in bold. Asterisks indicate haplotypes that have been previously identified in other horse breeds [26].

## Data Availability

Not applicable.

## Supplementary Materials

The following supporting information can be downloaded at: https://www.mdpi.com/article/10.3390/ani12080984/s1, Supplementary Material S1: Table S1.1. Primers used for gene expression analysis by RT-qPCR; Figure S1.1. Gene expression RT-qPCR; Figure S1.2. Surface markers expression; Figure S1.3. Results from the differentiation assays. Supplementary Material S2: Table S2.1. Antibodies used in panel 1 for flow cytometry assays; Table S2.2. Antibodies used in panel 2 for flow cytometry assays; Figure S2.1. Flow cytometry gating strategy.
