# Supplementary material for: Equine Mesenchymal Stem Cells Influence the Proliferative Response of Lymphocytes: Effect of Inflammation, Differentiation and MHC-Compatibility
Source: Animals (Basel). 2022 Apr 11;12(8):984. doi: 10.3390/ani12080984 (PMC9031781; doi:10.3390/ani12080984)
Supplement: Supplementary file 1 [file animals-12-00984-s001.zip › Supplementary material 1. Characterization Mesenchymal Stem Cells.pdf]

Supplementary material 1

Characterization of equine bone marrow derived mesenchymal stem cells (BM-MSCs).

1. Methodology

After their isolation, cells were characterized at passage 3 by the expression of defined markers and their ability to differentiate into adipocytes, osteoblasts and chondrocytes. Expression of positive markers CD90, CD105, CD44 and CD73 and lack of expression of the negative markers CD34 and CD45 was evaluated by real time quantitative polymerase reaction (RT-qPCR), and the surface expression of CD90 and CD105 was also studied by flow cytometry. In addition, surface expression of MHC-I and MHC-II was studied by flow cytometry in both MSC-naïve and MSC-primed since inflammatory exposure can induce changes in MHC expression.

1.1 Gene expression (RT-qPCR):

Isolation of mRNA and complementary DNA (cDNA) retrotranscription were performed using Cell-to-cDNA II (Ambion) according to the manufacturer's instructions and RT-qPCR reactions were performed and monitored with a QuantStudio3 Real Time PCR System device (Applied Biosystems). All reactions were carried out in a total volume of 10 μL with 2 μL of cDNA as the template and Fast SYBR Green Master Mix (Applied Biosystems). Amplification was performed in triplicate for each sample as follows: 20′′ at 95 °C for initial activation, followed by 40 cycles consisting of 3′′/95 °C and 30′′/60 °C and a dissociation curve protocol run after every PCR reaction. The levels of gene expression were determined by the comparative Ct method. Normalization factor was calculated as the geometric mean of the quantity of two housekeeping genes, GAPDH and B2M. Genes analysed, and corresponding primer sequences were previously designed by our group [13] and are presented in Table S1.1.

Table S1.1. Primers used for gene expression analysis by RT-qPCR.

Gene	*Accession number*	*Primer sequence (5′–3′)*	*Amplicon size (bp)*
		Housekeeping	
GAPDH	NM_001163856	F: GGCAAGTTCCATGGCACAGT<br>R: CACAACATATTCAGCACCAGCAT	128
B2M	NM_001082502.2	F: TCGTCCTGCTCGGGCTACT<br>R: ATTCTCTGCTGGGTGACGTGA	102
		Characterization cell surface markers	
		Mesenchymal cell markers	
CD90	EU881920	F: TGCGAACTCCGCCTCTCT<br>R: GCTTATGCCCTCGCACTTG	93
CD105	XM_001500078	F: GACGGAAAATGTGGTCAGTAATGA<br>R: GCGAGAGGCTCTCCGTGTT	100
CD44	NM 001085435	F: CCCACGGATCTGAAACAAGTG<br>R: TTCTGGAATTTGAGGTCTCCGTAT	95
CD73	XM 001500115	F: GGGATTGTTGGATACACTTCAAAAG<br>R: GCTGCAACGCAGTGATTTCA	90
		Hematopoietic markers	
CD34	XM_001491596	F: CACTAAACCCTCTACATCATTTTCTCCTA<br>R: GGCAGATACCTTGAGTCAATTTCA	150
CD45	AY_114350	F: TGATTCCCAGAAATGACCATGTA<br>R: ACATTTTGGGCTTGTCCTGTAAC	100

GenBank accession numbers of the sequences used for primers design. Primers (F: Forward and R: Reverse) and length of the amplicon in base pair (bp). Genes were grouped in agreement with the functions and implications of encoded molecules. GAPDH, Glyceraldehyde 3-phosphate dehydrogenase; B2M, beta-2 microglobulin; CD90, Thy-1; CD105, endoglin; CD44, hyaluronate receptor; CD73; CD34, haematopoietic marker; CD45, haematopoietic marker.

1.2 Surface markers expression (Flow cytometry):

Briefly, the cells were suspended in PBS/2 mM EDTA at 10^6cells/mL. 50 µL aliquots of cells were transferred to FACS tubes and separately incubated for 15 min at 4 °C in the dark with mouse anti-human monoclonal antibodies for CD90-PE (clone 5E10, BDPharmingen), CD105-FITC (clone SN6, R&D Systems), HLA-ABC-FITC (Beckman Coulter) and HLA-DR-APC (Immunostep). Anti-horse reactivity of antibodies was previously tested [13,29]. Subsequently, cells were washed with PBS (Gibco), diluted in 500 µL of PBS/2 mM EDTA and analysed with the Gallios flow cytometer (Beckman Coulter). Sytox Blue was used to gate live cells. Gating strategy was performed as described in [29] and data were analysed with FCS Express 7 Flow software (De Novo Software).

1.3 Tri-lineage differentiation:

The induction of differentiation into the osteogenic, adipogenic and chondrogenic lineages was performed by using specific media and successful differentiation was checked using specific staining and gene expression of lineage markers as previously described [15,17]. Each differentiation condition was assayed in triplicate for each donor (n=3), including non-differentiated controls cultured with regular media. For osteogenic, adipogenic and chondrogenic differentiation, cells were stained with 2 % Alizarin Red stain (pH 4.6), 0.3 % Oil-Red stain and 1 % Alcian Blue stains, respectively.

2. Results

2.1 Gene expression and surface markers expression:

Gene expression results showed that isolated MSCs from the three horses expressed CD90, CD105 and CD73 and did not express CD45, according to that established by the International Society for Cellular Therapy as minimal criteria for defining human MSCs [28]. However, CD34 expression was also observed in spite of being considered a negative marker (Figure S1.1). Nevertheless, phenotype variability among species has been described and CD34 gene expression has been previously observed in equine MSCs [27]. These MSCs were also positive for the typical MSC surface markers CD105 and CD90 determined by flow cytometry (Figure S1.2).

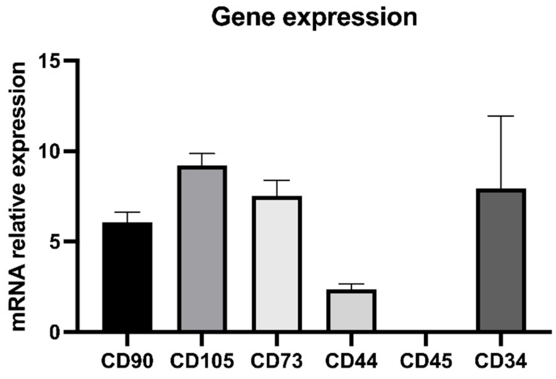

Figure S1.1. Data obtained from characterization assays using RT-qPCR are presented as mean ± S.E.M (n=3) of the mRNA relative expression of CD90, CD105, CD73, CD44, CD34 and CD45.

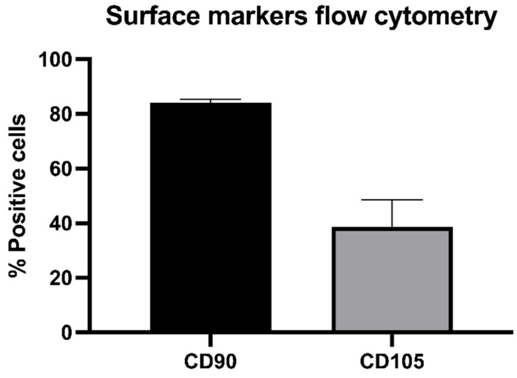

Figure S1.2. Mean ± S.E.M (n=3) of the percentage of positive cells for the surface markers CD90 and CD105 studied by flow cytometry.

2.2 Tri-lineage differentiation:

The tri-lineage differentiation ability was confirmed in equine MSCs (Figure S1.3). Specific staining for each differentiation assay resulted in calcium deposits for osteogenic differentiation (Figure S1.3.A), lipid droplets formation for adipogenic induction (Figure S1.3.B), and lacunae formation and proteoglycans production for chondrogenic differentiation (Figure S1.3.C). Mesenchymal stem cells used as control did not spontaneously differentiate (Figure S1.3.D).

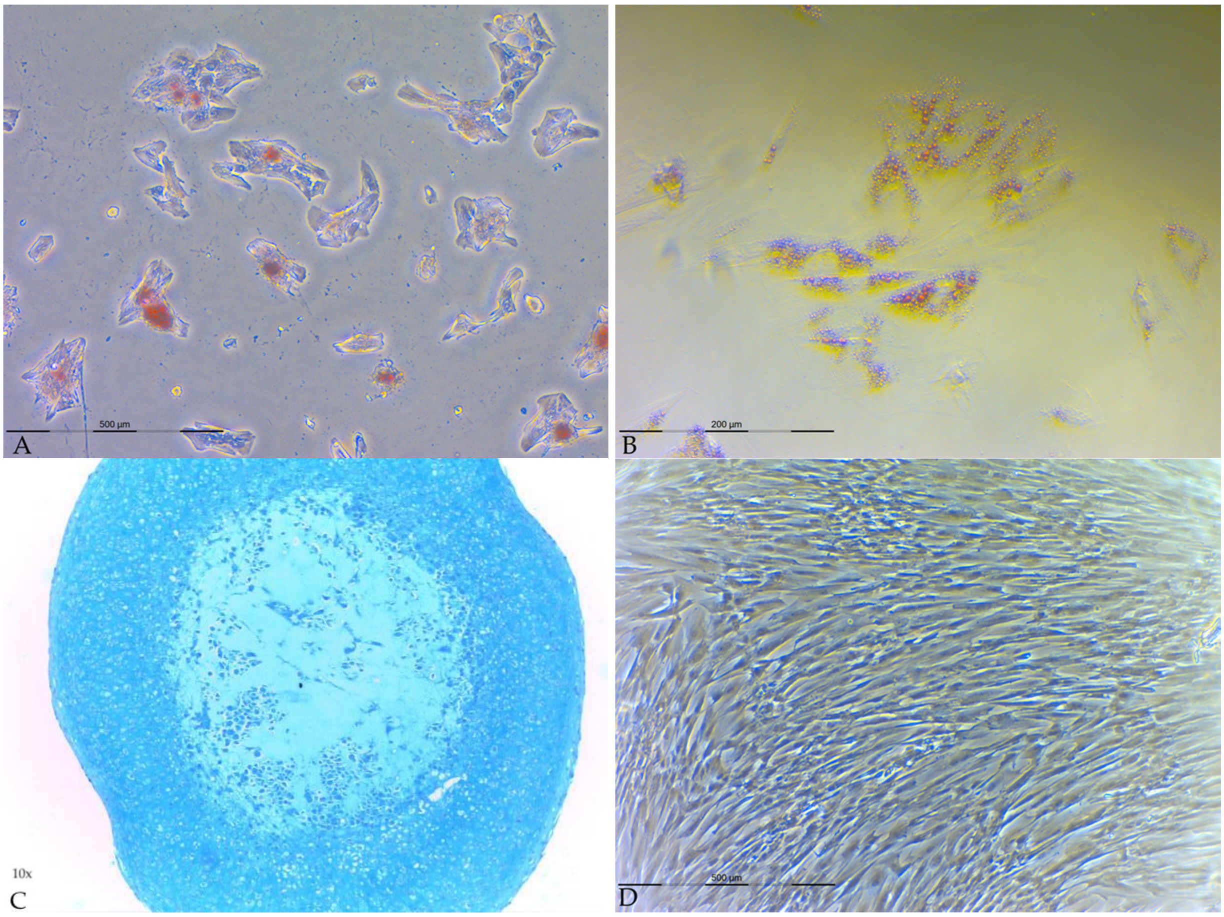

Figure S1.3. Results from the differentiation assays: calcium deposits stained by Alizarin Red in the cells undergoing osteogenesis (A), lipid droplets stained by Oil-O Red in the adipogenesis (B), proteoglycan rich extracellular matrix stained by Alcian Blue with lacunae formation after chondrogenic induction in 3D (pellet) culture (C) and undifferentiated control (D).
