# Supplementary material for: Equine Mesenchymal Stem Cells Influence the Proliferative Response of Lymphocytes: Effect of Inflammation, Differentiation and MHC-Compatibility
Source: Animals (Basel). 2022 Apr 11;12(8):984. doi: 10.3390/ani12080984 (PMC9031781; doi:10.3390/ani12080984)
Supplement: Supplementary file 1 [file animals-12-00984-s001.zip › Supplementary material 2. Antibodies and Flow cytometry.pdf]

Supplementary Material 2

Table S2.1. Antibodies used in panel 1 for flow cytometry assays

	Antibody	Host	Target species	Clone	Type and isotype	Conjugated and Fluorochrome	Dilution	Gallios cytometer channel	Target cells	References
PANEL 1	**Pan B-cells** MCA1899 Bio-Rad	Mouse	Anti-horse	CVS36	Monoclonal IgG1	Unconjugated (Surface staining)	1:100	-	B lymphocytes	[40,45]
	**Alexafluor™ 700 Goat anti-Mouse IgG Secondary Antibody** A-21036 Invitrogen	Goat	Anti-mouse	-	Policlonal IgG H+L	Alexa Fluor™ 700 Secondary antibody for anti-Pan B-cells	1:200	FL-7		
	**CD21** 561357 BD Pharmigen	Mouse	Anti-human	B-ly4	Monoclonal IgG1	APC (Surface staining)	1:40	FL-6	B lymphocytes	[44,45]
	**CD3** MCA1477 Bio-Rad	Rat	Anti-human (reacts with horse, bovine, pig and more)	CD3-12	Monoclonal IgG1	Unconjugated (Intracellular staining)	1:100	-	T lymphocyte	[4,34,41]
	**PE Mouse anti-Rat IgG1 Secondary Antibody** 12-4812-82 Invitrogen	Mouse	Anti-rat	R1-12D10	Monoclonal IgG1	PE Secondary antibody for anti-CD3	1:100	FL-2		

Table S2.2. Antibodies used in panel 2 for flow cytometry assays

	Antibody	Host	Target species	Clone	Type and isotype	Conjugated and Fluorochrome	Dilution	Gallios cytometer channel	Target cells	References
**PANEL 2**	**CD4** MCA1078 Bio-Rad	Mouse	Anti-horse	CVS4	Monoclonal IgG1	Unconjugated (Surface staining)	1:200		Helper T cells	[4,32,41,43,45]
	**APC/Cyanine7 Rat anti-mouse IgG1 Secondary Antibody** 406619 BioLegend	Rat	Anti-mouse	RMG1-1	Monoclonal IgG1	APC/Cyanine7 tandem Secondary antibody for anti-CD4	1:200	FL-8		
	**CD25/IL-2R** AF-223 R&D Systems	Goat	Anti-human	-	Polyclonal IgG	Unconjugated (Surface staining)	1:50		Regulatory T cells	[42,44,46,48]
	**Alexa Fluor® 647 Affini Pure Donkey Anti-Goat IgG (H+L)** 705-605-003 Jackson Immuno Research	Donkey	Anti-goat	-	Polyclonal IgG (H+L)	Alexa Fluor 647 Secondary antibody for anti-CD25:	1:400	FL-6		[44]
	**CD8** MCA2385 Bio-Rad	Mouse	Anti-horse	CVS8	Monoclonal IgG1	RPE (Surface staining)	1:5 (H2Od)	FL-2	Cytotoxic T cells	[45]

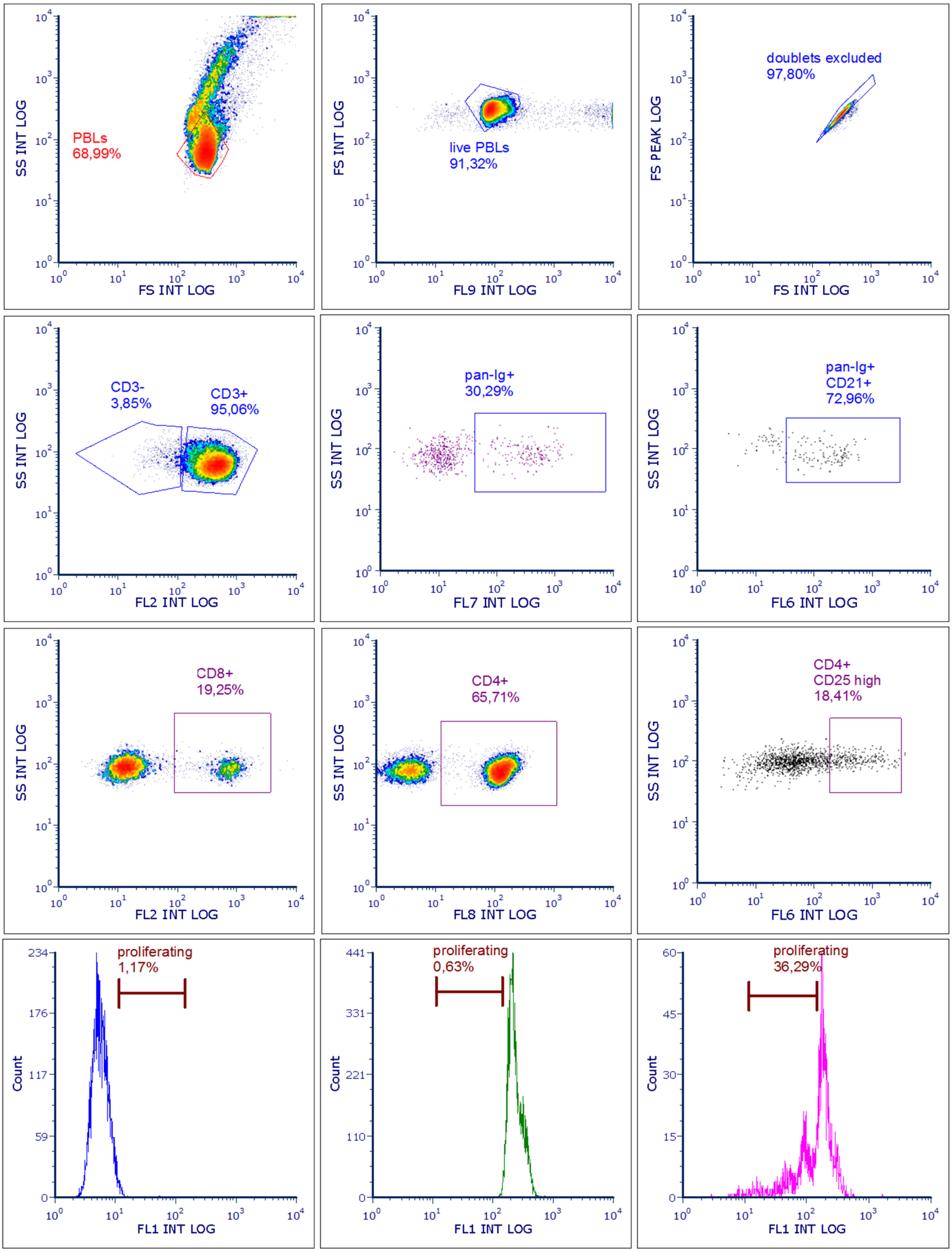

Figure S2.1. Flow cytometry gating strategy. First row shows how the population of lymphocytes (PBLs) was gated in the forward and side scatter (FSC x SSC) plot, and dead cells (FSC x FL9) and doublets (FS int x FS peak) were subsequently excluded. Second row represents gating of subpopulations analysed in panel 1: T cells as CD3+ (FL2) and B cells as CD3-/pan-Ig+/CD21+ (FL2/FL7/FL6). Third row presents gating for populations in panel 2: cytotoxic and helper T cells were gated as CD8+/CD4- and CD4+/CD8-, respectively (FL2/FL8), and regulatory T cells (Treg) were gated as CD4+/CD25^{high} (FL8/FL6). Fourth row: to study the proliferation of each subpopulation, CFSE dilution was assessed in FL1 using unstimulated and unstained PBLs to define the autofluorescence (left, blue histogram) and unstimulated and CFSE-labelled PBLs to set the non-proliferating population (middle, green histogram). Histogram in the right (pink) represents PBLs in the proliferating gate.
